# A novel multifunctional peptide oligomer of bacitracin with possible bioindustrial and therapeutic applications from a Korean food-source *Bacillus* strain

**DOI:** 10.1371/journal.pone.0176971

**Published:** 2017-05-11

**Authors:** Yun Hee Choi, Seung Sik Cho, Jaya Ram Simkhada, Md. Saifur Rahman, Yoon Seok Choi, Chun Sung Kim, Jin Cheol Yoo

**Affiliations:** 1 Department of Pharmacy, College of Pharmacy, Chosun University, Gwangju, Korea; 2 Department of Pharmacy, College of Pharmacy, Mokpo National University, Muan, Jeonnam, 5, Korea; 3 Department of Oral Biochemistry, College of Dentistry, Chosun Universit Gwangju, Korea; Emory University School of Medicine, UNITED STATES

## Abstract

Investigating the effects of a multifunctional microbial peptide possessing strong anti-inflammatory activity against pathogenic bacteria. The antimicrobial activity of the purified peptide (CSP32) against various multidrug-resistant as well as anaerobic pathogens was determined. Anti-inflammatory activity was determined by an enzyme-linked immunosorbent assay, western blotting, and RT-PCR in RAW 264.7 macrophages. Molecular weight and structural elucidation were performed by several analytical methods such as mass spectrometry and chemoinformatic analysis. CSP32, purified from newly isolated *Bacillus sp*. *CS32*, was active against methicillin-resistant *Staphylococcus aureus*, vancomycin-resistant *S*. *aureus*, vancomycin-resistant enterococci, and anaerobic pathogens *Propionibacterium acne* and *Clostridium difficile*. Furthermore, CSP32 showed strong inhibitory effects on lipopolysaccharide (LPS)-induced nitric oxide (NO) production and nitric oxide synthase (iNOS) and cyclooxygenase 2 (COX-2) expression in RAW 264.7 macrophages. At concentrations of 10, 50, and 100 μg/mL, CSP32 treatment attenuated LPS-induced expression of nuclear factor kappa B (NF-κB) and mitogen-activated protein kinases (MAPKs) as well as other proinflammatory mediators such as tumor necrosis factor α (TNF-α), interleukin 6 (IL-6), and IL-1β. CSP32 potently inhibited translocation of NF-κB into the nucleus by suppressing degradation of IκB kinase (IκBα) and its phosphorylation, thereby causing NF-κB to remain inactive. CSP32 may be the first oligomer of bacitracin with anti-inflammatory properties.

**Conclusion:**

CSP32 has stable characteristics and may find bio-industrial and therapeutic applications.

## Introduction

Antimicrobial peptides (AMPs) are ubiquitously secreted by a broad range of microorganisms, including bacteria, to protect themselves from other microbes [1]. These peptides are composed of 10–40 amino acid residues and affect active components of the innate immune response. In addition, AMPs have been confirmed to kill Gram-negative and positive bacteria including clinically important pathogens, mycobacteria, protozoa, viruses, fungi, and cancer cells [2]. AMPs produced by microbes such as *Bacillus* spp. have major therapeutic applications [3]. *Bacillus* is a genus of Gram-positive, rod-shaped, endospore-forming bacteria widespread in the environment and can serve as obligate aerobes or facultative anaerobes. *Bacillus* has been widely used in the fermentation industry for the production of antibiotics and several extracellular enzymes [4, 5]. A large number of peptides with biological activities from this group has been reported and has become a center of attention for antimicrobial research [3, 6–14]. On the basis of their origin, these peptides are categorized into two classes: nonribosomally synthesized and ribosomally synthesized. The first group includes bacitracin, penicillin, and some others, containing nonprotein amino acids such as D-amino acids or hydroxyl amino acids and other amino acids that undergo extensive modification. The second group, which includes subtilin, undergoes posttranslational modification and proteolytic processing [10].

Inflammation is a part of the complex biological response of vascular tissues to harmful stimuli, such as pathogens, damaged cells, or irritants [15]. The inflammatory process is a protective response to infection, tissue injury, or noxious stimuli [16]. MAPKs translate extracellular signals into a variety of cellular processes, including cell differentiation, proliferation, survival, and apoptosis. NF-κB is one of the most vital transcription factors involved in transactivation of a variety of genes associated with regulation of immune function, cellular proliferation, inflammatory responses, and tumorigenesis. In the inflammatory process, activated inflammatory cells secrete increased amounts of nitric oxide (NO) and cytokines, such as interleukins IL-1β, IL-6, and tumor necrosis factor (TNF)-α [17]. NO is a major product, and its formation is controlled by NO synthases (e.g., iNOS). iNOS is highly expressed in macrophages, and its activation leads to organ destruction in some inflammatory and autoimmune diseases. During inflammation, macrophages play a central role in the management of many immunopathological phenomena, including the overproduction of proinflammatory cytokines and inflammatory mediators such as iNOS, COX-2, TNF- α, IL-1β, and IL-6 [17, 18].

The rapid development of antibiotic resistance has drawn many researchers’ attention to the search for new antibacterial agents. Nonsteroidal anti-inflammatory drugs (NSAIDs) have been used widely in our society for the treatment of acute and chronic inflammatory diseases. However, treatment with NSAIDs for a prolonged period is associated with a major side effect: gastrointestinal problems. Therefore, researchers have continued to show interest in screening of new biological compounds from various sources. For a long time, our research group has maintained interest in identification of new compounds that have both antibacterial and anti-inflammatory properties with low toxicity.

With the aim of characterizing a microbial peptide with therapeutic applications, an AMP-producing strain was isolated from traditional fermented Korean kimchi. The AMP was purified, biochemically characterized, and its structure was analyzed. The antimicrobial and anti-inflammatory activities of the pure compound were evaluated.

## Materials and methods

### Materials

Sepharose CL-6B and Sephadex G-50 were acquired from Pharmacia (Uppsala, Sweden). Trypsin, proteinase K, α-chymotrypsin, and lipase were purchased from Sigma (St. Louis, MO, USA). Lipopolysaccharide (LPS), dimethyl sulfoxide (DMSO), Griess reagent, and 3-(4,5-dimethylthiazol-2-2,5-diphenyltetrazolium bromide (MTT) were also purchased from Sigma. Dulbecco’s modified Eagle’s medium (DMEM), fetal bovine serum (FBS), and a penicillin-streptomycin solution were acquired from Invitrogen (Grand Island, NY, USA). A rabbit anti-mouse iNOS polyclonal antibody was purchased from Santa Cruz Biotech Inc. (Santa Cruz, CA, USA), and an anti-mouse COX-2 antibody from Cayman Co. Horseradish peroxidase (HRP)-conjugated donkey anti-rabbit IgG antibody and anti-mouse IgG antibody were purchased from Cell Signaling Technology (Danvers, MA). Alkaline-phosphatase-conjugated donkey anti-mouse IgG antibody was purchased from Jackson Immunoresearch Laboratories Inc. Mouse TNF-α, IL-6, IL-1β, and an enzyme-linked immunosorbent assay (ELISA) kits were purchased from BD Biosciences (San Diego, CA, USA). All other chemicals and reagents were of analytical grade.

### *In vitro* screening, isolation, and culture conditions for *Bacillus sp*. *CS32*

Korean fermented kimchi food samples were collected at various locations in Chonnam Province. The Korean food samples were resuspended in distilled water. After dilution, the samples were inoculated onto the surface of *Bacillus* isolation agar (MRS, Mueller Hinton) plates. The 16S ribosomal RNA (rRNA) sequence analysis was carried out according to Bergey’s *Manual of Systemic Bacteriology* as stated in our previous report [19]. All the non-ATCC strains are deposited in Korean collection for type culture (KCTC), which is belong to World Data Centre for Microorganisms (WDCM). AMP production by the strain was optimized by means of several carbon and nitrogen sources. The influence of various carbon sources on AMP production was determined using media supplemented with 1% yeast extract combined with 1% supplements such as glucose, mannitol, starch, lactose, fructose, sorbitol, sucrose, or maltose. Fermentation was carried out in 250-mL Erlenmeyer flasks containing 50 mL of a medium with constant shaking at 180 rpm. After that, the influence of the nitrogen source on peptide production was determined using a medium containing 1% glucose (best carbon source) combined with 1% supplements such as beef extract, malt extract, tryptone, yeast extract, oat meal, soytone, or peptone.

### Purification of CSP32

*Bacillus* sp. CS32 was cultured for 60 h in the glucose–beef extract–peptone medium (1% glucose, 0.5% beef extract, and 0.5% peptone). The cells were separated by centrifugation (6000 × g rpm, 30 min, 4°C), and peptides were precipitated from the supernatant at 4°C overnight with ammonium sulfate of 60% saturation. The precipitate was collected by centrifugation (6000 × g rpm, 60 min, 4°C), resuspended in 10 mM Tris-HCl buffer (pH 8.0) and dialyzed using a 1-kDa cutoff membrane (Millipore). The crude extracts were applied to a Sepharose CL-6B column (2.2 × 116 cm) and next to a Sephadex G-50 column (1.5 × 70 cm), and eluted with the same buffer. Active fractions were pooled and concentrated using a YM1 amicon filter and stored at −20°C.

### Analysis of molecular weight and N-terminal amino acid sequence

The molecular weight of the purified peptide was determined by Tricine-sodium dodecyl sulfate-polyacrylamide gel electrophoresis (tricine SDS-PAGE). For *in situ* detection of inhibitory activity, the gel was washed with 50 mM Tris-HCl buffer (pH 7.9) containing 2.5% Triton X-100 and overlaid with soft agar containing an indicator strain, *Mycobacterium smegmatis* ATCC 9341, (10^6^ colony-forming units) and incubated overnight at 37°C. N-terminal amino acid sequencing was carried out in the Process 492 Amino Sequencer (Applied Biosystems, Foster City, CA) by the automated Edman degradation method.

### Mass spectrometry (MS) and chemoinformatic analysis

The molecular mass of the peptide was evaluated by matrix-assisted laser desorption ionization-time-of-flight (MALDI-TOF) MS. Furthermore, liquid chromatography with tandem mass spectrometry (LC-MS/MS) was performed on a triple-quadruple tandem mass spectrometer in positive electrospray ionization (ESI) mode as well. The product mass spectra were collected in the m/z range from 50 to 3000 Da.

### Antimicrobial activity of CSP32

This activity in terms of minimal inhibitory concentration (MIC) was determined by dilution and disk diffusion methods as per the Clinical and Laboratory Standards Institute (CLSI) guidelines for antimicrobial susceptibility testing [20]. The examination of bacteria was conducted after incubation for 18 h at 37°C following inoculation of various test microorganisms, including methicillin-resistant *Staphylococcus aureus (MRSA)*, *vancomycin-resistant S*. *aureus (VRSA)*, or *vancomycin-resistant enterococci (VRE)*. Control antibiotics were bacitracin and vancomycin.

Susceptibilities for *Propionibacterium acnes KCTC5933* and *Clostridium difficile KCTC2251* were determined using the NCCLS reference agar dilution procedure [21]. A colony from a 40 h culture was suspended in thioglycolate broth and adjusted a density equivalent to a McFarland 0.5 standard then inoculated onto Brucella agar (Difco, Becton Dickinson, Sparks MD21152, USA) supplemented with 5% lysed sheep blood, 5 mg/L haemin and 1 mg/L vitamin K. Afterwards, plates were inoculated with ~10^5^ cfu per spot, using an antibiotic sensitivity replicator (H.I. Clements Pty Ltd., Sydney, Australia). All plates were incubated in an anaerobic cabinet (Don Whitley Scientific Ltd, West Yorkshire, England) for 48 h. *Propionibacterium acnes* and *Clostridium difficile* were used an anaerobic control bacteria. All antimicrobials were tested at dilutions, from 0.2 to 0.02 mg/L (CSP32) and 1 to 0.125 mg/L (Monensin).

### Stability of the *Bacillus sp*. *CS32*-produced AMP

To analyze thermostability, CSP32 activity was evaluated by measuring residual activities after various treatments. After heat treatment (30–121°C, 10–60 min), the samples were tested for residual antimicrobial activity against indicator strain *Micrococcus luteus* ATCC 9341. Effects of pH on antimicrobial activity were estimated by varying the pH levels. Samples were diluted in the following buffers: citrate with Na_2_HPO_4_ (pH 3.0–7.0), Tris-HCl (pH 7.0–9.0), and NaHCO_3_ with NaOH (pH 10.0–11.0). After incubation for 1 h at room temperature, pH in the samples was adjusted to 7.0, and the samples were tested for antimicrobial activity against the indicator strain. The AMP was digested with various proteolytic enzymes at a final concentration of 1 mg/mL: lipase (50 mM Tris-HCl, pH 7.5), protease K (50 mM Tris-HCl, pH 7.5), α-chymotrypsin (50 mM Tris-HCl, pH 7.5), or trypsin (50 mM Tris-HCl, pH 8.0). After 1-h incubation at room temperature, the enzymes were boiled for 2 min for inactivation.

### Cell culture and assessment of cell viability

Cell viability was assessed using the 3-(4,5-dimethylthiazol-2-yl)-2,5-diphenyltetrazolium bromide (MTT) assay as described previously by Choi et al. (2016) [22]. For the cell viability assay, RAW 264.7 cells were seeded into a 96-well flat bottom microplate at a density of 2 × 104 cells per well and incubated at 37°C for 1 h. The cells were then treated with various concentrations (5–100μg/mL) of CSP32. After 24 h of incubation, the medium was removed and wash in PBS once followed by 100 μl of MTT (5 mg/ml in PBS) solution was added to each well, and the plate was incubated for another 1 h. Then 100 μl of DMSO (100%) was added to dissolve the formazan crystals. Absorbance values were then measured at 450 nm using a microplate reader (Victor3, PerkinElmer).

### NO analysis and quantification of cytokine production

NO levels in the culture supernatants were measured by the Griess reaction. Cells (10^6^ cells/mL) were seeded in 6-well plates and pretreated with the indicated concentrations of AMP for 30 min before stimulation with 1 μg/mL LPS for 24 h. The sample supernatants were mixed with an equal volume of the Griess reagent (1% sulfanilamide in 5% phosphoric acid and 0.1% naphthyl ethylene diamine dihydrochloride), and then incubated at room temperature for 10 min. The absorbance was measured at 550 nm on a microplate reader (Thermo Fisher Co.). The nitrite concentration was determined using a dilution of sodium nitrite as a standard.

### An enzyme immunoassay of cytokines

The inhibitory effects of CSP32 on the production of proinflammatory cytokines—TNF-α, IL-1β, and IL-6—were determined by an enzyme-linked immune sorbent assay (ELISA). Cells were incubated with various concentrations of CSP32 (10–100 μg/mL) for 1 h followed by LPS (1 μg/mL) treatment for 24 h. The concentration of nitrite in the supernatant was calculated using a sodium nitrite standard curve. The supernatant was analyzed for TNF-α, IL-1β, and IL-6 by ELISAs using commercial kits (BD Biosciences) according to the manufacturer’s instructions.

### Western blotting

Macrophages were incubated with or without LPS in the presence or absence of the AMPs. Cells were harvested, washed twice with ice-cold Tris-HCl-buffered saline (TBS), and resuspended in lysis buffer [100 mM Tris-HCl, 5 mM EDTA, 50 mM NaCl, 50 mM β- glycerophosphate, 50 mM NaF (sodium fluoride), 0.5% NP-40, 1% sodium deoxycholate, 0.1 mM sodium orthovanadate, and 1% phenylmethylsulfonyl fluoride (PMSF)]. The cytosolic fraction was obtained from the supernatant after 12000 × g rpm centrifugation at 4°C for 20 min. Samples (20 μg in terms of protein) were separated by sodium dodecyl sulfate-polyacrylamide gel electrophoresis (SDS-PAGE) in a 10% gel and transferred to polyvinylidene difluoride (PVDF) membranes. The membranes were blocked with 5% nonfat milk in TBS with Tween 20 (0.1%) for 2 h and then incubated with primary antibodies specific for ERK, phospho-ERK, JNK, phospho-JNK, p38 MAPK, phospho-p38 MAPK (all them from Cell Signaling Technology), NF-κB, IκB, and phospho-IκB (1:1000; Santa Cruz Biotechnology). To prove equal loading, the blots were analyzed for β-actin expression using an anti-β-actin antibody (Santa Cruz Biotechnology). After three washes with TBS–Tween 20, the membrane was hybridized with an HRP-conjugated secondary antibody for 1 h. The membranes were washed three times for 10 min and developed with the ECL Western Blotting Detection System (GE Healthcare, Incheon, Republic of Korea,). The immunoreactive proteins were detected by means of a LAS-3000 Luminescent image analyzer (Fuji Photo Film Co., Ltd., Tokyo, Japan).

### RNA preparation and mRNA expression analysis by reverse-transcription (RT)-PCR

Total RNA from the AMP-treated cells was prepared using the RNAiso Reagent (Takara) and was stored at −80°C until use. For the detection of iNOS, COX-2, TNF-α, IL-Iβ, and IL-6, total RNA was extracted after stimulation and treatment. The mRNA expression levels of iNOS, COX-2, TNF-α, IL-Iβ, and IL-6 in the treated cells were compared to the expression levels in control cells. One microgram of RNA was reverse-transcribed into cDNA and used as a template for PCR amplification. The primer sequences are listed in Table 1. PCR was performed on a DNA Gene Cycler, under the following conditions: 40 cycles of denaturation at 94°C for 30 s, annealing at 58°C for 30 s, and primer extension at 72°C for 40 s. PCR products were analyzed on 1% agarose gels, and bands were visualized by ethidium bromide staining.

**Table 1:** Primers for RT-PCR.

Primers	Primer sequences	
	Forward	Reverse
iNOS	5′-CCCTTCCGAAGTTTCTGGCAGCAGC-3′	5′-GGCTGTCAGAGCCTCGTGGCTTTGG-3′
COX-2	5′-CACTACATCCTGACCCACTT-3′	5′-ATGCTCCTGCTTGAGTATGT-3′
TNF-α	5′-TCTCATCAGTTCTATGGCCC-3′	5′-GGGAGTAGACAAGGTACAAC-3′
IL-1β	5′-TGGACGGACCCCAAAAGATG-3′	5′-AGAAGGTGCTCATGTCCTCA-3′
IL-6	5′-GTTCTCTGGGAAATCGTGGA-3′	5′-TGTACTCCAGGTAGCTATGG-3′
GAPDH	5′-CACTCACGGCAAATTCAACGGCAC-3′	5′-GACTCCACGACATACTCAGCAC-3′

### Statistical analysis

Student’s *t*-test and one- way multivariate analysis of variance (ANOVA) were used to analyze the data. Differences were considered significant when *p < 0.01.

## Results

### Identification of strain CS32

**Fig 1:**
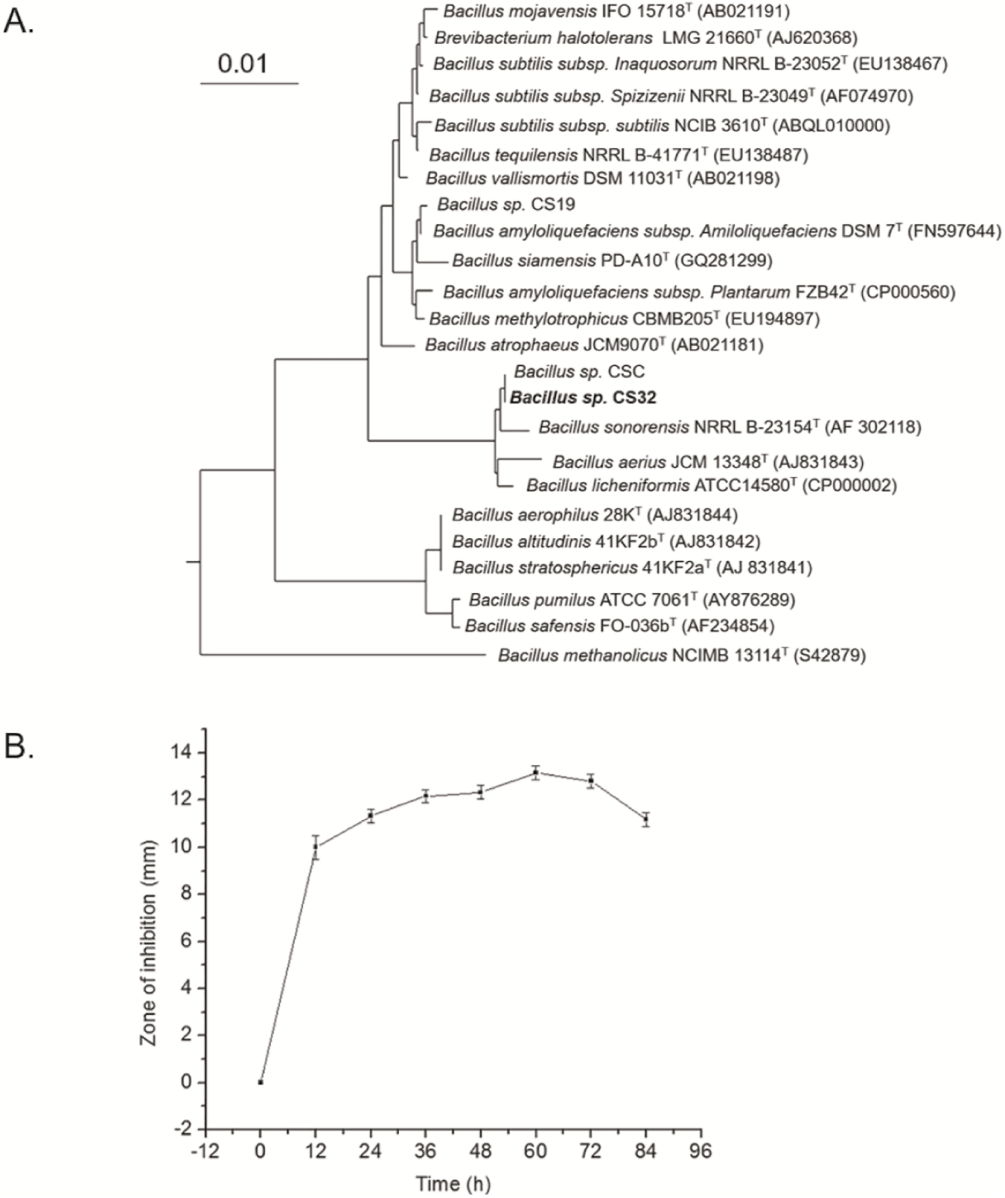
(A) A neighbor-joining tree based on nearly complete 16S rRNA gene sequences showing relations between strain CS32 and related members of the genus *Bacillus*. The percentages at the nodes are the levels of bootstrap support based on neighbor-joining analyses of 1000 resampled datasets. The sequence of *Bacillus licheniformis* ATCC 14580(T) served as an outgroup. The scale bar: 0.01 nucleotide substitutions per position. (B) Production of CSP32 and the growth-inhibitory activity against *Mycobacterium smegmatis* ATCC 9341. The antibacterial activity is maximal at 60 h. The zone of inhibition against *Mycobacterium smegmatis* ATCC 9341 at every 12 h is shown. Cultivation was carried out in 250-mL flasks with 50 mL of a medium, at pH 7 and 37°C, with shaking at 180 rpm.

Microbial strains capable of producing AMPs were isolated from traditional Korean fermented kimchi. Morphological and biochemical characteristics showed that strain CS32 is a *Bacillus* species. Furthermore, the 16S rRNA sequence of the local isolate was compared with the sequences of closely related *Bacillus* species. Computer-assisted RNA searches in a bacterial database revealed that the 16S rRNA sequence of CS32 was 99.392% identical to *Bacillus licheniformis*. The phylogenetic tree based on the sequence alignment is presented in Fig 1A.

### Purification, molecular weight and N-terminal sequence analysis

**Fig 2:**
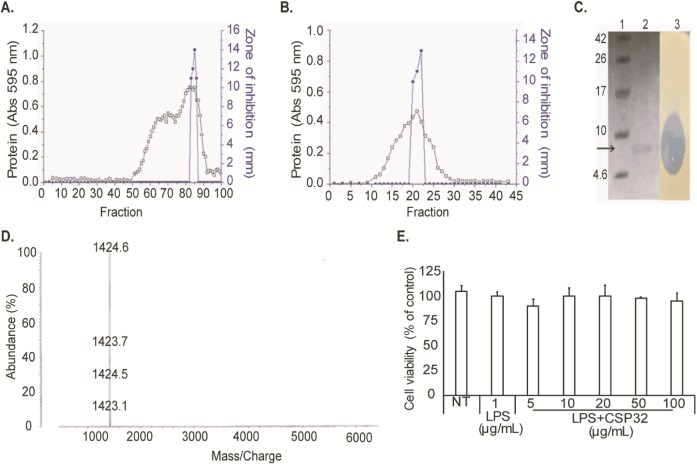
(A) Gel filtration chromatography on a Sepharose CL-6B column (2.2 cm × 116 cm). The proteins were eluted at a flow rate of 5 mL/min. (B) Gel filtration chromatography on a Sephadex G-50 column (1.5 cm × 70 cm). The proteins were eluted at a flow rate of 1 mL/min. Determination of the molecular weight. (C) Tricine SDS-PAGE and activity staining of CSP32. Tricine SDS-PAGE: Lane 1, protein molecular weight markers with the corresponding value in kDa on the left; Lane 2, purified CSP32; Lane 3, activity staining (*in situ*). (D) MALDI-TOF-MS. (E) Cell viability was measured after 24-h incubation. Survival rates were tested by an MTT assay in RAW 264.7 cells. The cells were incubated in the presence or absence of 5–100 μg/mL CSP32 for 24 h. Each bar shows mean ± SD of three independent experiments performed in triplicate.

The CSP32, from 60 h cultured cell-free supernatant (Fig 1B), was purified to homogeneity by a two-step procedure (**Fig 2A and 2B**). Tricine SDS-PAGE and an assay of *in situ* inhibitory activity (Fig 2C) confirmed the homogeneity and activity of the purified AMP. In the MALDI-TOF-MS analysis (Fig 2D), CSP32 appeared as a single peak with the molecular mass of 5697.9 Da which confirmed the presence of single compound. The first 12 amino acid residues of the N terminus of CSP32 were found to be APLEIXXIFHDN. The sequence was compared with related bacitracin derivatives presented in Table 2.

**Table 2:** Comparative analysis of N-terminal amino acid sequences.

Sl. No.	peptide	amino acid residues	theoretical MW (Da)	identity (%)	monomer sequence
1	CSP32	A-P-L-E-I-X-X-I-F-H-D-N	1390.98	100	**current study**
2	Bacitracin A1	I-C-L-E-I-K-O-I-F-H-D-N	1581.89	66.7	Position 1: Ile, position 2: Cys
3	Bacitracin A2	I-C-L-E-I-K-O-I-F-H-D-N	1581.89	66.7	1: D-aIle, 2: Cys
4	Bacitracin D1	V-C-L-E-I-K-O-V-F-H-D-N	1553.84	66.7	2: Cys
5	Bacitracin H1	V-C-L-E-I-K-O-I-F-H-D-N	1567.87	66.7	2: dhCys
6	Bacitracin F	I-C-L-E-I-K-O-I-F-H-D-N	1581.89	66.7	1: Ile, 2: dhCys
7	Bacitracin B3	I-C-L-E-I-K-O-V-F-H-D-N	1567.87	58.3	2: CYS
8	Bacitracin I1	V-C-L-E-I-K-O-V-F-H-D-N	1553.84	58.3	2: dhCys
9	Bacitracin I2	V-C-L-E-V-K-O-I-F-H-D-N	1553.84	58.3	2: dhCys
10	Bacitracin H2	I-C-L-E-I-K-O-V-F-H-D-N	1567.87	58.3	2: dhCys
11	Bacitracin H3	I-C-L-E-V-K-O-I-F-H-D-N	1567.87	58.3	2: dhCys
12	Bacitracin B2	I-C-L-E-V-K-O-I-F-H-D-N	1567.87	58.3	2: Cys
13	Bacitracin B1	V-C-L-E-I-K-O-I-F-H-D-N	1567.87	58.3	2: Cys
14	Bacitracin D2	V-C-L-E-V-K-O-I-F-H-D-N	1553.84	58.3	2: Cys
15	Bacitracin I3	I-C-L-E-V-K-O-V-F-H-D-N	1553.84	50.0	2: dhCys
16	Bacitracin E	V-C-L-E-V-K-O-V-F-H-D-N	1539.81	50.0	-
17	Bacitracin C	I-C-L-E-V-K-O-V-F-H-D-N	1553.84	50.0	2: Cys

### The effect of CSP32 on viability of RAW 264.7 cells

RAW 264.7 cells were incubated with various concentrations of CSP32 for 24 h and cell viability was assessed by the MTT assay. As shown in Fig 2E, CSP32 did not exert cytotoxicity toward RAW 264.7 cells in the range of 5–100 μg/mL. Therefore, CSP32 was used at or below 100 μg/mL in the further experiments.

### Structure elucidation

**Fig 3:**
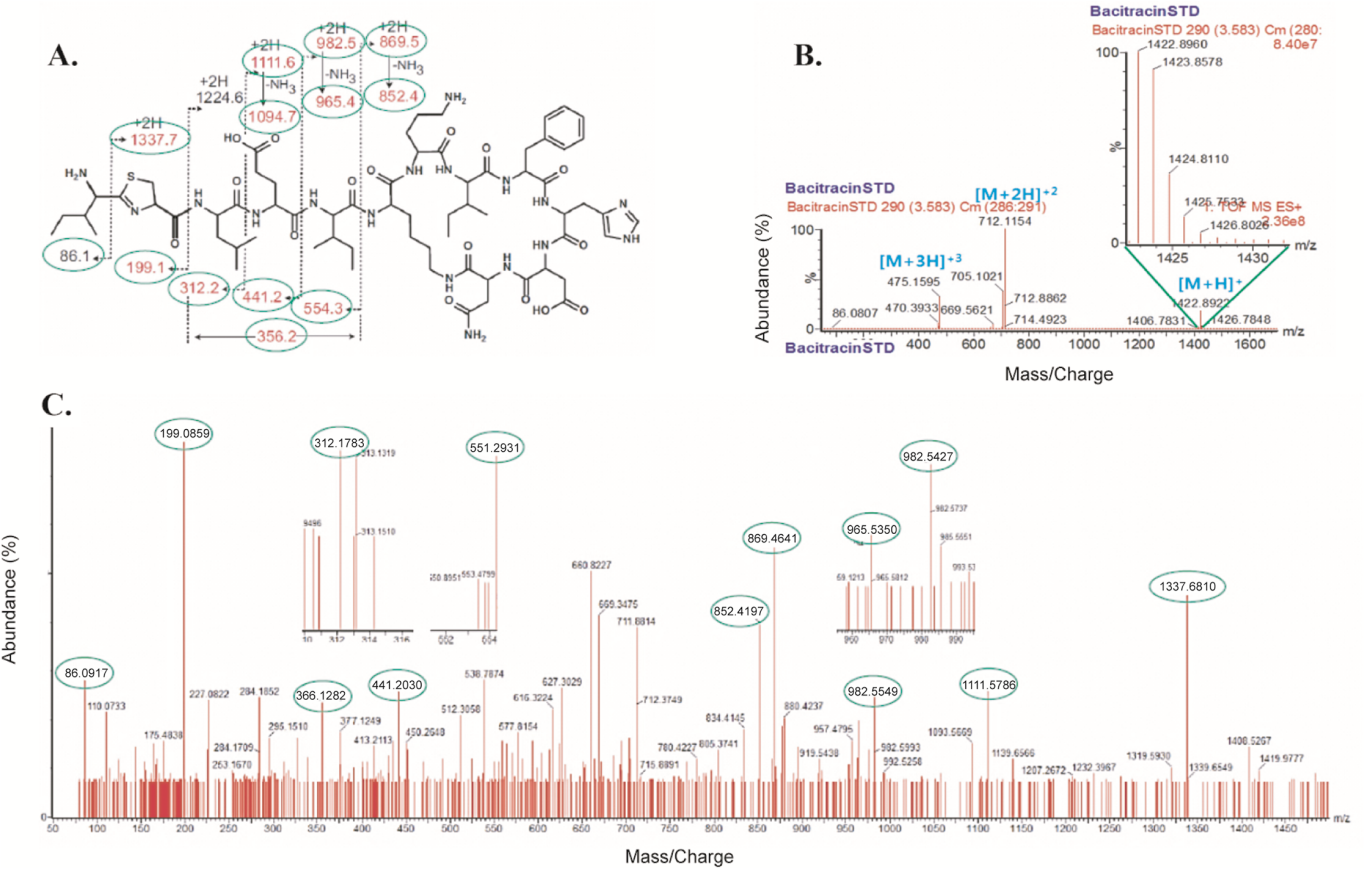
Chemoinformatic analysis of bacitracin A. (A) Standard structure of bacitracin A; (B) fragmentation patterns and percentage of abundance of standard bacitracin A and (C) of CSP32.

The MS result of CSP32 matched with Bacitracin ~1422 Da with several subunits (up to 11). A robust and efficient HR-QTOF ESI/MS analysis revealed the existence of a complex of 1–11 monomers of bacitracin, a nonribosomal peptide (Table 3), in CSP32. MS/MS analysis of CSP32 and peptide sequencing (analysis of the MS fragmentation pattern) showed that the MS/MS molecular pattern was identical to that of bacitracin A (Fig 3A–3C).

**Table 3:** Comparison of MS data with predicted molecular weight of each subunit.

Subunit no	1	2	3	4	5	6	7	8	9	10	11
MW X unit no	1421.7540	2843.5080	4265.2620	5687.0160	7108.7700	8530.5240	9952.2780	11374.0320	12795.7860	14217.5400	15639.2940
	1421.7540	2843.5080	4265.1894	5687.0004	7108.7504	8530.4875	9952.3705	11374.9940	12795.7555	14217.5000	15639.5010
	1422.7567	2844.5112	4266.1544	5688.0012	7109.7520	8531.4920	9953.3330	11375.9935	12796.8480	14218.9380	15641.3730
	1423.7597	2845.5134	4267.1876	5689.0125	7110.7780	8532.5310	9954.3035	11377.0030	12797.8370	14220.3040	15642.2640
	1424.7615	2846.5202	4268.1876	5690.0178	7111.7648	8533.5055	9955.2755	11378.0345	12798.8245	14221.4610	15642.9816
	1425.7600	2847.5194	4269.1832	5691.0183	7112.7676	8534.5250	9956.2780	11379.0225	12799.8475	14222.5820	15644.2674
MS data	1426.7638	2848.5144	4270.1958	5692.0143	7173.7696	8535.5270	9957.2820	11380.0415	12800.7530	14223.5890	15645.1848
	1427.7617	2849.5230	4271.1768	5693.0234	7114.7636	8536.5390	9958.2815	11381.0290	12801.8360		15646.3302
	1428.7636	2850.5250	4272.2246	5694.0237	7115.7656	8537.5115	9959.2770	11382.0645	12802.8200		15647.2176
	1429.7592	2851.5200		5695.0125	7116.7712	8538.5200	9960.2960	11383.0520	12803.7915		15648.3324
	1430.7570	2852.5114		5696.0238	7118.7848	8539.5195	9961.2915	11384.0550	12804.8265		15649.2642
	1431.7538	2853.5276		5697.0195	7119.7876	8540.5480	9963.2830	11384.9965	12805.8315		15650.2926
	1432.7393						9964.3160	11387.0445	12806.7895		15651.3954
								11388.0590	12807.8030		15652.3416
									12809.9095		15653.2998
											15654.4032
											15655.4178
											15656.2356

### Antimicrobial activity of CSP32

As shown in Table 4, CSP32 showed antimicrobial activity against Gram-positive but not Gram-negative bacteria. Moreover, CSP32 exerted an antagonistic effect against multidrug-resistant pathogens such as *M*. *luteus*, *MRSA*, *VRSA*, and *VRE*, which was similar or in some cases stronger than that of bacitracin or vancomycin.

**Table 4:** Minimum inhibitory concentration of CSP32 against various pathogens.

Test organisms	MIC (μg/mL)		
	CSP32	Bacitracin	Vancomycin
*Alcaligenes faecalis* ATCC 1004	>80	>80	>80
*Salmonella typhimurium* KCTC 1925	>80	>80	>80
*Escherichia coli* KCTC 1923	>80	>80	>80
*Pseudomonas aeruginosa* KCTC 1637	>80	>80	>80
*Micrococcus luteus* ATCC 9341	0.156	>80	0.156
*Mycobacterium smegmatis* ATCC 9341	>80	>80	0.3125
*Enterococcus faecalis* ATCC 29212	5	5	2.5
*Bacillus subtilis* ATCC 6633	>80	40	>80
*Listeria monocytogenes* KCTC3569	10	0.3125	0.156
*Staphylococcus aureus* KCTC 1928	5	40	0.3125
MRSA 639E	2.5	40	0.3125
MRSA 4–5	5	1.25	0.3125
MRSA 5–3	2.5	1.25	0.3125
MRSA U4	40	1.25	0.3125
MRSA S3	10	1.25	0.3125
MRSA P3	40	1.25	0.625
MSRA S1	40	1.25	0.625
VRSA	40	>80	>80
VRE 2	10	0.3125	>80
VRE3	>80	20	>80
VRE4	10	5	>80
VRE5	10	1.25	>80
VRE6	>80	>80	>80
VRE82	80	40	>80
VRE89	>80	40	>80

Furthermore, CSP32 showed potent antimicrobial activity against anaerobic pathogens *P*. *acne* and *C*. *difficile*. When we compared its activity with monensin, CSP32 exerted manyfold stronger activity than that of monensin against those pathogens (Table 5).

**Table 5:** Antimicrobial activity of CSP32 and Monensin against anaerobic pathogenic microorganisms.

	Concentration (mg/mL)	*Propionibacterium acnes (mm)*	*Clostridium* *difficile (mm)*
CSP32	0.2	23.33±0.58	19.00±0.50
	0.1	19.00±0.50	17.17±0.29
	0.04	17.33±0.58	15.17±0.29
	0.02	12.83±0.29	13.17±0.58
Monensin	1	17.17±0.29	17.33±0.29
	0.5	17.33±0.58	15.17±0.29
	0.25	15.17±0.29	13.00±0.50
	0.125	13.00±0.50	13.17±0.29

Results are presented as means ± standard deviation (n = 3).

### Thermal, pH, and proteolytic resistance of CSP32

The effects of temperature and pH on stability of CSP32 are shown in Table 6. These data indicate that CSP32 was almost completely stable up to 90°C and at pH 5.0–12.0. Its activity decreased sharply at or above 121°C. To test the effect of proteolytic enzymes, CSP32 was incubated with lipase, proteinase K, α-chymotrypsin, and trypsin agar diffusion assay. Residual activity in the presence of lipase, proteinase K, α-chymotrypsin or trypsin was 98.3%, 95.7%, 94.3%, and 100%, respectively, as compared to no-treatment control (set to 100%) (data not provided). Results are presented as means ± standard deviation (n = 3).

**Table 6:** Thermo and pH-stability of CSP32.

Temperature	Time	Residual activity (%)
**None**		100
**30°C**	10 min	100
	30 min	100±0.28
	60 min	100±0.34
**50°C**	10 min	100±0.94
	30 min	98.40±0.39
	60 min	98.07±0.36
**70°C**	10 min	98.07±0.41
	30 min	98.00±0.41
	60 min	98.07±0.39
**90°C**	10 min	97.73±1.04
	30 min	95.93±0.05
	60 min	95.93±0.05
**121°C**	15 min	65.40±0.17
**pH**		
**pH 2**		61.33±1.04
**pH 3**		63.00±1.04
**pH 4**		89.60±3.44
**pH 5**		95.87±2.29
**pH 6**		100±0.50
**pH 7**		100
**pH 8**		100±0.20
**pH 9**		100±1.15
**pH 10**		98±3.38
**pH 11**		91.33±0.06
**pH 12**		91.77±0.06

Results are presented as means ± standard deviation (n = 3)

### Inhibition of NO production and iNOS, COX-2 protein expression in LPS-stimulated RAW 264.7 macrophage cells

**Fig 4:**
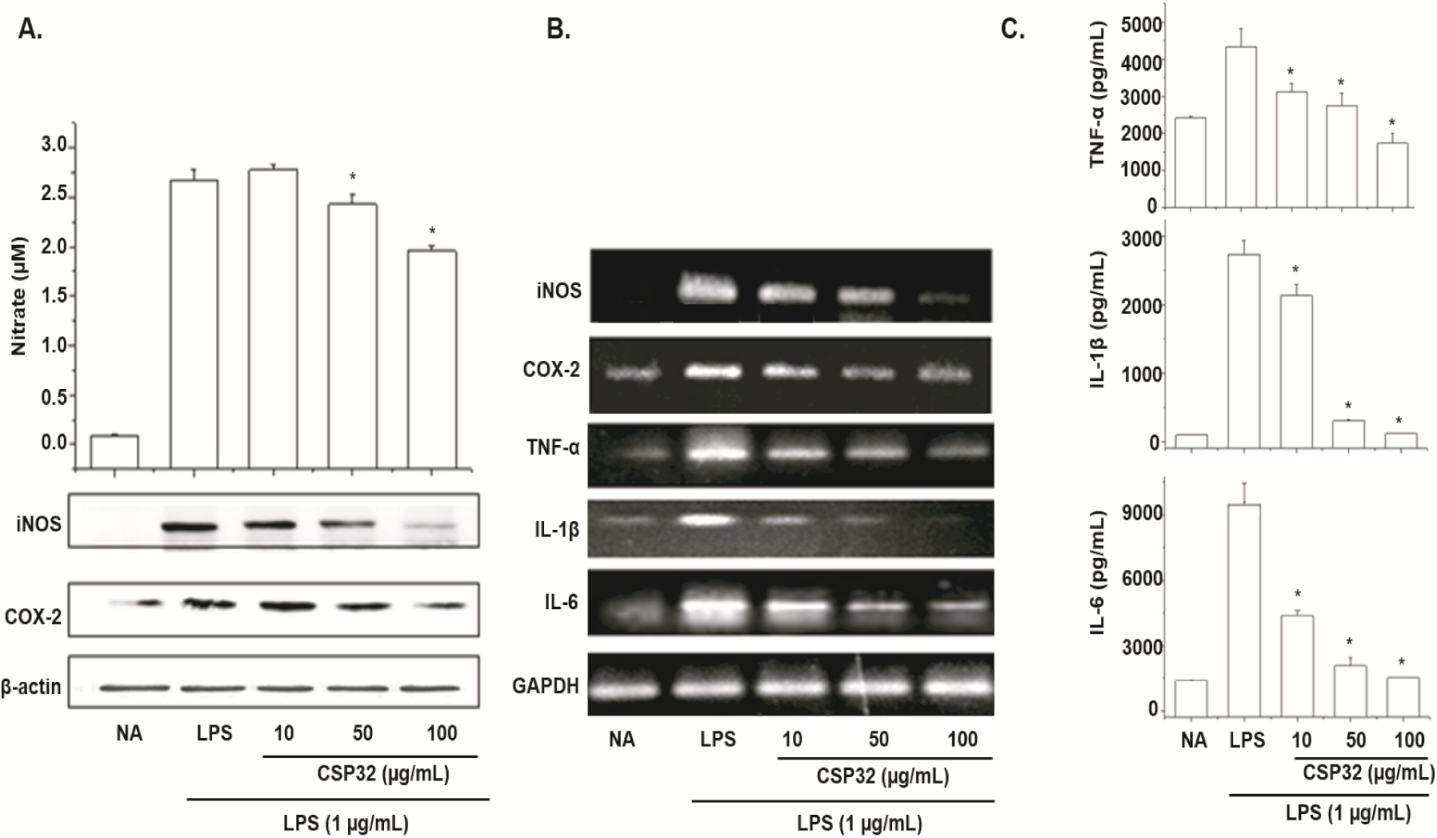
Effects of CSP32 on NO production and expression of inflammation-associated proteins in LPS-stimulated RAW 264.7 macrophage cells. The cells were treated with LPS (1 μg/mL) in the presence of various concentrations of CSP32 (10, 50, or 100 μg/mL) at 37°C for 24 h. (A) The amounts of NO were determined using the Griess reagent in the culture medium (Top panel). Equal amounts of cell lysates were resolved on SDS polyacrylamide gels, transferred to PVDF membranes, and probed with antibodies against iNOS and COX-2. β-Actin served as an internal control for western blot analysis (bottom panel). Effects of CSP32 on LPS-induced mRNA expression of iNOS, COX-2, TNF-α, IL-1β, and IL-6. (B) After LPS treatment for 2–12 h, the levels of iNOS, COX-2, TNF-α, IL-1β, and IL-6 mRNA were determined by RT-PCR. *GAPDH* served as an internal control for RT-PCR assays. Suppressive effects of CSP32 on TNF-α, IL-1β, and IL-6 production in LPS-stimulated RAW 264.7 macrophage cells. (C) The cells were incubated with the indicated concentrations of CSP32 for 30 min before treatment with LPS (1 μg/mL) for 24 h. After incubation for 24 h, the supernatant was collected, and the amounts of these proinflammatory cytokines were measured by ELISAs. Results represent the mean±S.D of three independent experiments performed in triplicate. * p<0.01; compared to LPS alone.

To assess the effect of CSP32 on NO production by LPS-stimulated RAW 264.7 cells, we measured NO concentration in the culture medium by the Griess method [23]. As shown in Fig 4A, LPS treatment significantly increased NO production as compared to the untreated cells. Treatment of cells with CSP32 at 10, 50, or 100 μg/mL attenuated the LPS-induced production of NO to a statistically significant extent.

In an attempt to determine whether the inhibition of NO production by CSP32 was related to downregulation of iNOS and COX-2, we next examined the protein expression levels by western blotting. As shown in Fig 4A, both iNOS and COX-2 protein levels increased in the LPS-stimulated cells when compared to the controls. CSP32 treatment significantly attenuated this induction in a dose-dependent manner. The suppression of iNOS and COX-2 protein expression correlated well with that of NO production.

### Effects of CSP32 on NO production and expression of inflammation-associated proteins in LPS-stimulated RAW 264.7 macrophage cells

The cells were treated with LPS (1 μg/mL) in the presence of various concentrations of CSP32 (10, 50, or 100 μg/mL) at 37°C for 24 h. (A) The amounts of NO were determined using the Griess reagent in the culture medium (Top panel). Equal amounts of cell lysates were resolved on SDS polyacrylamide gels, transferred to PVDF membranes, and probed with antibodies against iNOS and COX-2. β-Actin served as an internal control for western blot analysis (bottom panel). Effects of CSP32 on LPS-induced mRNA expression of iNOS, COX-2, TNF-α, IL-1β, and IL-6. (B) After LPS treatment for 2–12 h, the levels of iNOS, COX-2, TNF-α, IL-1β, and IL-6 mRNA were determined by RT-PCR. *GAPDH* served as an internal control for RT-PCR assays. Suppressive effects of CSP32 on TNF-α, IL-1β, and IL-6 production in LPS-stimulated RAW 264.7 macrophage cells. (C) The cells were incubated with the indicated concentrations of CSP32 for 30 min before treatment with LPS (1 μg/mL) for 24 h. After incubation for 24 h, the supernatant was collected, and the amounts of these proinflammatory cytokines were measured by ELISAs. Results represent the mean±S.D of three independent experiments performed in triplicate. * p<0.01; compared to LPS alone.

### Inhibition of mRNA expression of iNOS, COX-2, TNF-α, IL-1β, and IL-6 in LPS-stimulated RAW 264.7 macrophage cells

To determine whether the above effect on NO production was related to changes in the levels of iNOS and COX-2, we measured the expression of iNOS and COX-2 mRNA by RT-PCR (primers are shown in Table 1). Glyceraldehyde 3-phosphate dehydrogenase (GAPDH) was used to normalize gene expression data. In the cells treated with LPS, the AMP significantly attenuated iNOS and COX-2 mRNA upregulation in a dose-dependent manner. We also examined mRNA expression of several proinflammatory cytokines, such as TNF-α, IL-1β, and IL-6. LPS treatment increased TNF-α, IL-1β, and IL-6 mRNA levels and treatment of cells with CSP32 dose-dependently attenuated the LPS-enhanced expression (Fig 4B).

### Inhibitory effects of CSP32 on the production of proinflammatory cytokines in LPS-stimulated RAW 264.7 macrophage cells

LPS-stimulated macrophages promote inflammation by secreting proinflammatory cytokines such as TNF-α, IL-1β, and IL-6. To test whether CSP32 affects cytokine production in LPS-stimulated RAW 264.7 cells, we measured TNF-α, IL-1β, and IL-6 concentrations in culture supernatants by ELISAs. LPS treatment significantly increased TNF-α, IL-1β, and IL-6 production as compared to the untreated cells. As shown in Fig 4C, the LPS-induced release of cytokines was significantly attenuated by CSP32 in a dose-dependent manner. These results revealed that CSP32 blocked LPS-induced proinflammatory mediators such as TNF-α, IL-1β, and IL-6 in macrophages. These results suggest CSP32 as a potential anti-inflammatory agent.

### Effects of CSP32 on LPS-induced phosphorylation of MAPKs

**Fig 5:**
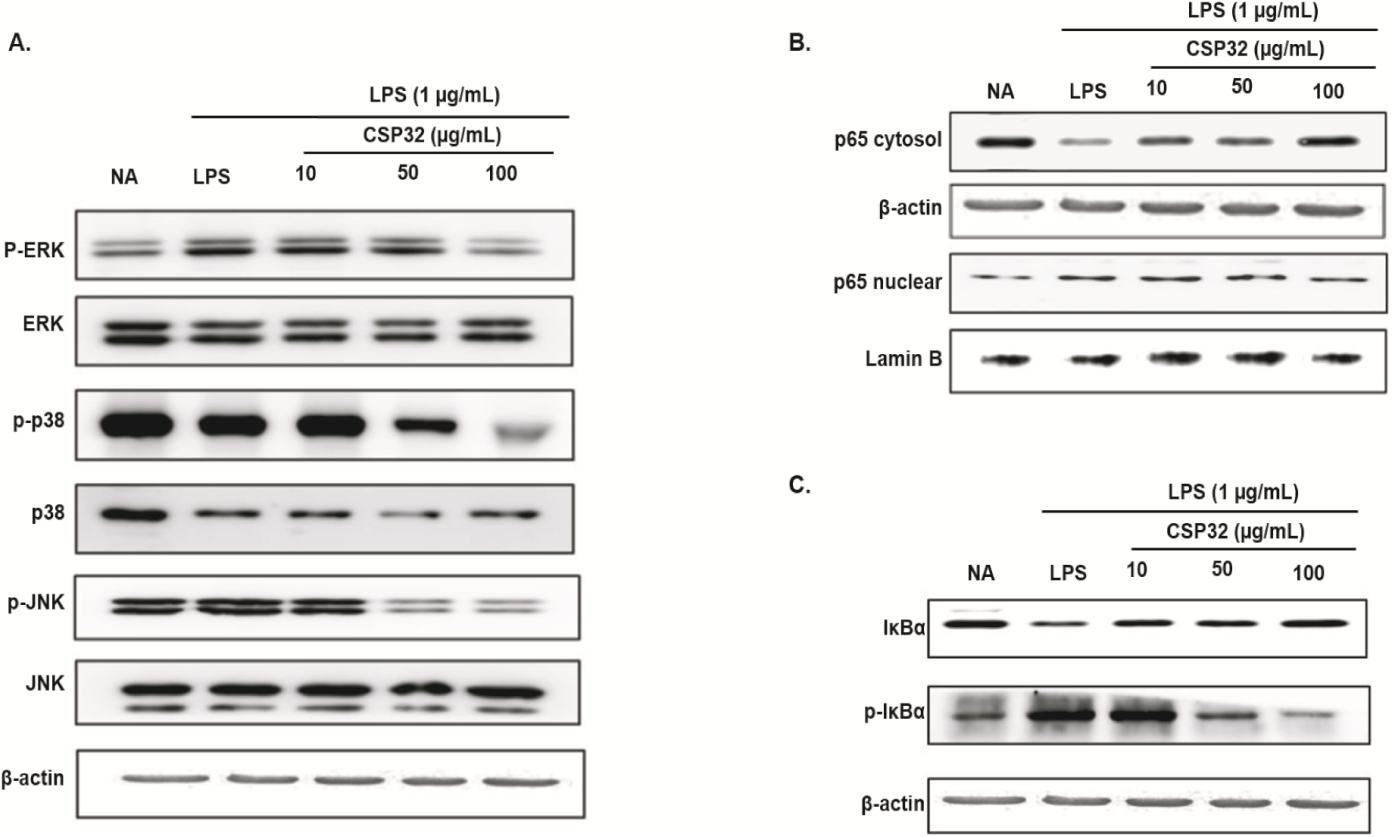
(A) Effects of CSP32 on phosphorylation of MAPKs (ERK, JNK, and p38) in the LPS-stimulated RAW 264.7 cells. Effects of CSP32 on LPS-stimulated NF-κB expression, to be precise, inhibition of translocation into the nucleus (B) and inhibition of IκBα phosphorylation and degradation (C). The cells were separately treated with the indicated concentrations of CSP32 for 1 h prior to the addition of 1 μg/mL LPS, and the cells were further incubated for 24 h.

As shown in Fig 5A, phosphorylation of ERK, JNK, and p38 MAPKs increased significantly when the cells were treated with LPS (1 μg/mL) for 1 h. Phosphorylation decreased in a dose-dependent manner by the treatment with CSP32 (10–100 μg/mL). In contrast, the amounts of unphosphorylated ERK, JNK, and p38 MAPKs were unaffected by the treatment with LPS or CSP32.

### Effects of CSP32 on NF-κB translocation and on phosphorylation of IκBα in LPS-stimulated RAW 264.7 macrophage cells

To assess the effects of CSP32 on LPS-induced NF-κB activation, we examined the translocation of NF-κB into the nucleus using western blot analysis. As shown in Fig 5B, cytoplasmic levels of p65 decreased in response to LPS treatment. The LPS-induced changes in the levels of p65 in the nucleus and cytoplasm were attenuated in macrophages treated with CSP32. Furthermore, the level of p-IκBα that was increased by LPS was attenuated by CSP32 treatment (dose dependently), and at the same time, the total level of IκBα in the cytoplasm increased (dose dependently) as shown in Fig 5C. This finding suggests that an increase of CSP32 dose enhanced the ability of IκBα to maintain NF-κB in an inactive form in the cytoplasm.

## Discussion

The primary aim of this study was to isolate, identify, and characterize a potent substance that has antimicrobial and anti-inflammatory properties from a local fermented food source (kimchi). Microbial strains capable of producing AMPs were isolated from Korean traditional food kimchi. Strain CS32 was selected from the various screened strains because of its well-pronounced antimicrobial activity. Based on morphological and physiological parameters and 16S rRNA gene sequences, the strain was identified as *Bacillus* sp. CS32.

This strain shares the highest 16S rRNA homology (99.392%) with *B*. *licheniformis*, which was reported to produce the antibacterial agent bacitracin. The AMP (designated as CSP32) produced by the strain in the optimal medium was purified to homogeneity and was found to have a molecular mass of ~5700 Da (~5.7 kDa). The first 12 amino acid residues of CSP32 are APLEIXXIFHDN. The molecular mass, together with the distinct N-terminal amino acid sequence, makes CSP32 a novel antimicrobial peptide. In addition, CSP32 showed a strong structural similarity of its monomeric sequence to the 12-amino acid sequence of bacitracin A, a derivative of bacitracin, and the chemoinformatic analysis confirmed this result.

Bacitracin is an antibiotic produced by certain species, including *B*. *licheniformis* and *B*. *subtilis*. According to Bonsai: Bioinformatics Software Server (http://bioinfo.lifl.fr/), only 16 bacitracin derivatives have been identified to date. The FHDN motif is common in all bacitracin derivatives, including CSP32. According to an NCBI BLAST search, the sequence of CSP32 shows the highest homology to a bacteriocin-like compound (lichenin) from *B*. *licheniformis* [24]. Besides, the sequence also showed some degree of identity with AMPs from *B*. *subtilis* JM4 [10] with shared sequences EIxxIFHDN where x represents unidentified residues. CSP32 was found to be almost completely stable up to 90°C and at pH 5.0–12.0. The pH and temperature stability of CSP32 is comparable with that of lichenin and AMPs produced by the *B*. *subtilis* strain JM4 [10, 24]. CSP32 has molecular weight 1390.98 Da (theoretical) according to the first 12 amino acids, which have a high degree of similarity to bacitracin derivatives (monomeric peptides; Table 2), as determined by Edman degradation method. On the other hand, MALDI-TOF-MS showed molecular weight of 5697.90 Da for a complex of four peptides (consisting of 12 amino acid residues). We determined the structural similarity by comparing experimental ESI-MS/MS fragmentation of CSP32 and spectra of fragments derived from bacitracin A. MS-MS analysis of CSP32 and peptide sequence analysis in the fragmentation pattern revealed that the MS-MS molecular masses of 199.1, 356.2, 441.1, 852.4, 982.5, 1111.6, and 1337.7 are similar to the fragmentation pattern of bacitracin A. In addition, the standard bacitracin A fragmentation analysis (molecular weights 475.1595, 7112.1154, and 1422.8960) showed a pattern similar to that of CSP32. Furthermore, the findings from HR-QTOF-ESI/MS revealed that the quaternary structure consists of 11 peptide subunits (Table 3). On the basis of the results of three different experiments, clearly CSP32 is an oligomer of bacitracin, and Edman degradation method was able to read only the first 12 amino acids of the N terminus. The single band in tricine SDS-PAGE corresponded to molecular weight 5697.90 Da from MALDI-TOF-MS results. Just as other well-known AMPs, CSP32 is not sensitive to all the proteases tested, suggesting that it is nonproteinaceous as previously reported [24, 25]. Although high proteolytic-enzyme concentrations were necessary to inactivate the antimicrobial activity, some antimicrobials produced by *Bacillus* spp. are cyclic peptides containing unusual amino acids, which are more resistant to proteolytic enzymes. CSP32 may be a novel peptide because of its unique N-terminal amino acid sequence along with other biochemical properties. Effects of CSP32 were evaluated against various pathogenic Gram-positive and negative bacteria. CSP32 exerted antimicrobial action on Gram-positive microorganisms, in agreement with the effects of antimicrobial peptides from *Bacillus* sp. JM4 [10] and *B*. *licheniformis* A89 [26].

Besides, the antimicrobial effect of CSP32 against multidrug-resistant bacteria such as *MRSA*, *VRSA*, and *VRE* was comparable with the action of bacitracin or vancomycin. Nevertheless, CSP32 is slightly less potent than BCP61 [25]. Moreover, CSP32 showed strong antimicrobial activity against anaerobic pathogens *P*. *acne* and *C*. *difficile*. When we compared its activity with that of monensin, CSP32 was more effective than monensin against those pathogens (**Table 5**). Monensin is a polyether ionophore widely used in veterinary medicine for the prevention and treatment of coccidiosis in poultry and as a growth promoter in ruminants [27]. Although it is popular in many countries, its residues in food may pose a risk to health among sensitive individuals [27, 28]. Our results show that CSP32 may be useful for the reduction of methane gas production and for enhancement of feed efficacy via management of anaerobic intestinal microorganisms.

In accordance with the second objective, this study revealed that CSP32 significantly attenuates LPS-induced production of proinflammatory mediators NO, iNOS, and COX-2 in RAW 264.7 cells. Moreover, CSP32 suppressed the activation MAPKs (ERK, JUN, and p38) and of NF-κB by LPS (Fig 5A), and our data are consistent with the following mechanism: p65 binding activity and CSP32 prevent IκBα phosphorylation and degradation; the effects of CSP32 on both phenomena are dose-dependent (Fig 5B and 5C). Our results indicate that the inhibitory effects of CSP32 are mediated by iNOS and COX-2 suppression. Furthermore, the RT-PCR analysis suggests that mRNA levels of iNOS and COX-2 correlate with their protein levels. Therefore, the inhibitory effect of CSP32 on iNOS and COX-2 expression appears to be one of the mechanisms responsible for the anti-inflammatory action of AMPs. Hence, CSP32 suppresses the expression of genes implicated in inflammation. We evaluated production of proinflammatory cytokines in LPS-stimulated cells, specifically TNF-α, IL-1β, and IL-6. These cytokines are produced mainly by activated macrophages. Suppression of cytokine production is an important way to counter the inflammatory process [15–17]. Because AMPs have been reported to downregulate proinflammatory mediators [24], we next investigated the effects of CSP32 on the LPS-induced release of cytokines by ELISAs and RT-PCR. There was no change in basal cytokine expression after incubation with only CSP32 without LPS. Incubation with only LPS strongly upregulated the proinflammatory cytokines. After 24-h incubation with both LPS (1 μg/mL) and CSP32, there was a remarkable attenuation of this increased cytokine production and mRNA expression in RAW 264.7 cells. These results suggest that CSP32 is a potent inhibitor of LPS-induced iNOS and COX-2 mRNA and protein expression and cytokine production in RAW 264.7 cells (Fig 4), in line with other reports [15–17].

We are currently conducting such experiments as evaluation of peptide analogs, and luciferase assays. To the best of our knowledge, an oligomer consisting of peptide subunits likely to be a bacitracin derivative, i.e., purified CSP32—which has strong antimicrobial and anti-inflammatory properties, and was isolated from *Bacillus sp*. *CS32* from fermented kimchi—has never been reported.

## Conclusion

CSP32 has potent antimicrobial and anti-inflammatory activities and was purified from a *Bacillus* strain newly isolated from traditional Korean fermented kimchi. CSP32 is likely to be a novel oligomer of a bacitracin derivative because of its unique N-terminal amino acid sequence along with other biochemical properties. Our results show that CSP32 may be a suitable candidate for the development of a multifunctional peptide for the prevention or treatment of infections caused by multidrug-resistant and anaerobic pathogens as well as inflammation-related disorders.

## References

[1] Cook RJ, Thomashow LS, Weller DM, Fujimoto D, Mazzola M, Bangera G, et al (1995) Molecular mechanisms of defense by rhizobacteria against root disease. Proc Natl Acad Sci U S A 92(10):4197–201. Epub 1995/05/09. PubMed Central PMCID: PMC41910. 11607544 10.1073/pnas.92.10.4197 PMC41910

[2] Rana M, Chatterjee S, Kochhar S, Pereira B (2006) Antimicrobial peptides: a new dawn for regulating fertility and reproductive tract infections. J Endocrinol Reprod 2:88–95.

[3] Bizani D, Brandelli A (2002) Characterization of a bacteriocin produced by a newly isolated Bacillus sp. Strain 8 A. J Appl Microbiol 93(3):512–9. Epub 2002/08/14. 12174052 10.1046/j.1365-2672.2002.01720.x

[4] Sumi CD, Yang BW, Yeo I-C, Hahm YT (2014) Antimicrobial peptides of the genus Bacillus: a new era for antibiotics. Canadian journal of microbiology 61(2):93–103. doi: 10.1139/cjm-2014-0613 25629960 10.1139/cjm-2014-0613

[5] Tamang JP, Watanabe K, Holzapfel WH (2016) Review: diversity of microorganisms in global fermented foods and beverages. Frontiers in microbiology 7. 10.3389/fmicb.2016.00377 PMC4805592 27047484

[6] Cherif A, Ouzari H, Daffonchio D, Cherif H, Ben Slama K, Hassen A, et al (2001) Thuricin 7: a novel bacteriocin produced by Bacillus thuringiensis BMG1.7, a new strain isolated from soil. Lett Appl Microbiol 32(4):243–7. Epub 2001/04/12. 11298934 10.1046/j.1472-765x.2001.00898.x

[7] Ahern M, Verschueren S, van Sinderen D (2003) Isolation and characterisation of a novel bacteriocin produced by Bacillus thuringiensis strain B439. FEMS Microbiol Lett 220(1):127–31. Epub 2003/03/20. 12644238 10.1016/S0378-1097(03)00086-7

[8] Dischinger J, Josten M, Szekat C, Sahl HG, Bierbaum G (2009) Production of the novel two-peptide lantibiotic lichenicidin by Bacillus licheniformis DSM 13. PLoS One 4(8):e6788 Epub 2009/08/27. Central PMCID: PMC2727956. doi: 10.1371/journal.pone.0006788 19707558 10.1371/journal.pone.0006788 PMC2727956

[9] Oscariz JC, Lasa I, Pisabarro AG (1999) Detection and characterization of cerein 7, a new bacteriocin produced by Bacillus cereus with a broad spectrum of activity. FEMS Microbiol Lett 178(2):337–41. Epub 1999/09/28. 10499284 10.1111/j.1574-6968.1999.tb08696.x

[10] Wu S, Jia S, Sun D, Chen M, Chen X, Zhong J, et al (2005). Purification and characterization of two novel antimicrobial peptides Subpeptin JM4-A and Subpeptin JM4-B produced by Bacillus subtilis JM4. Curr Microbiol 51(5):292–6. Epub 2005/10/08. doi: 10.1007/s00284-005-0004-3 16211432 10.1007/s00284-005-0004-3

[11] Lee HJ, Joo YJ, Park CS, Kim SH, Hwang IK, Ahn JS, et al (1999). Purification and characterization of a bacteriocin produced by Lactococcus lactis subsp. lactis H-559 isolated from kimchi. J Biosci Bioeng 88(2):153–9. Epub 2005/10/20. 16232590 10.1016/s1389-1723(99)80194-7

[12] Kayalvizhi N, Gunasekaran P. Production and characterization of a low-molecular-weight bacteriocin from Bacillus licheniformis MKU3 (2008) Lett Appl Microbiol 47(6):600–7. Epub 2009/01/06. doi: 10.1111/j.1472-765X.2008.02473.x 19120933 10.1111/j.1472-765X.2008.02473.x

[13] Khalil R, Elbahloul Y, Djadouni F, Omar S (2009) Isolation and partial characterization of a bacteriocin produced by a newly isolated Bacillus megaterium 19 strain. Pak J Nutr 8:242–50.

[14] Rosenfeld Y, Shai Y (2006) Lipopolysaccharide (Endotoxin)-host defense antibacterial peptides interactions: Role in bacterial resistance and prevention of sepsis. Biochimica et Biophysica Acta (BBA)—Biomembranes. 1758(9):1513–22. 16854372 10.1016/j.bbamem.2006.05.017

[15] Sarkar D, Saha P, Gamre S, Bhattacharjee S, Hariharan C, Ganguly S, et al (2008) Anti-inflammatory effect of allylpyrocatechol in LPS-induced macrophages is mediated by suppression of iNOS and COX-2 via the NF-kappaB pathway. Int Immunopharmacol 8(9):1264–71. Epub 2008/07/08. doi: 10.1016/j.intimp.2008.05.003 18602073 10.1016/j.intimp.2008.05.003

[16] Yoon WJ, Heo SJ, Han SC, Lee HJ, Kang GJ, Kang HK, et al (2012) Anti-inflammatory effect of sargachromanol G isolated from Sargassum siliquastrum in RAW 264.7 cells. Arch Pharm Res 35(8):1421–30. Epub 2012/09/04. doi: 10.1007/s12272-012-0812-5 22941485 10.1007/s12272-012-0812-5

[17] Yu HY, Kim KS, Lee YC, Moon HI, Lee JH (2012) Oleifolioside A, a New Active Compound, Attenuates LPS-Stimulated iNOS and COX-2 Expression through the Downregulation of NF-kappaB and MAPK Activities in RAW 264.7 Macrophages. Evid Based Complement Alternat Med 2012:637512 Epub 2012/08/23. PubMed Central PMCID: PMC3405816. doi: 10.1155/2012/637512 22911495 10.1155/2012/637512 PMC3405816

[18] Yoon WJ, Kim SS, Oh TH, Lee NH, Hyun CG. Cryptomeria japonica essential oil inhibits the growth of drug-resistant skin pathogens and LPS-induced nitric oxide and pro-inflammatory cytokine production (2009) Pol J Microbiol 58(1):61–8. Epub 2009/05/28. 19469288

[19] Cho SS, Choi YH, Simkhada JR, Mander P, Park da J, Yoo JC. A newly isolated Streptomyces sp. CS392 producing three antimicrobial compounds (2012) Bioprocess Biosyst Eng 35(1–2):247–54. Epub 2011/09/13. doi: 10.1007/s00449-011-0599-7 21909674 10.1007/s00449-011-0599-7

[20] Jorgensen JH, Turnidge JD (2015) Susceptibility test methods: dilution and disk diffusion methods Manual of Clinical Microbiology, Eleventh Edition: American Society of Microbiology p. 1253–73.

[21] National Committee for Clinical Laboratory Standards (2004) Methods for Antimicrobial Susceptibility Testing of Anaerobic Bacteria—Sixth Edition: Approved Standard M11-A6. NCCLS, Wayne, PA, USA.

[22] Choi YH, Na BH, Choi YS, Rahman MS, Kim MR, Jee J-P, et al (2016) Anti-inflammatory function of 4-tert-butylphenyl salicylate through down-regulation of the NF-kappa B pathway. Archives of pharmacal research 39(3):429–36. doi: 10.1007/s12272-015-0679-3 26849878 10.1007/s12272-015-0679-3

[23] Kim HK, Cheon BS, Kim YH, Kim SY, Kim HP. Effects of naturally occurring flavonoids on nitric oxide production in the macrophage cell line RAW 264.7 and their structure–activity relationships (1999) Biochem Pharmacol 58(5):759–65. 10449184 10.1016/s0006-2952(99)00160-4

[24] Pattnaik P, Kaushik JK, Grover S, Batish VK (2001) Purification and characterization of a bacteriocin-like compound (Lichenin) produced anaerobically by Bacillus licheniformis isolated from water buffalo. J Appl Microbiol 91(4):636–45. Epub 2001/09/29. 11576300 10.1046/j.1365-2672.2001.01429.x

[25] Choi YH, Cho SS, Simkhada JR, Yoo JC (2012) A novel thermotolerant and acidotolerant peptide produced by a Bacillus strain newly isolated from a fermented food (kimchi) shows activity against multidrug-resistant bacteria. Int J Antimicrob Agents 40(1):80–3. Epub 2012/05/15. doi: 10.1016/j.ijantimicag.2012.03.019 22578764 10.1016/j.ijantimicag.2012.03.019

[26] Mendo S, Faustino NA, Sarmento AC, Amado F, Moir AJ (2004) Purification and characterization of a new peptide antibiotic produced by a thermotolerant Bacillus licheniformis strain. Biotechnol Lett 26(2):115–9. Epub 2004/03/06. 15000477 10.1023/b:bile.0000012888.72489.3f

[27] Chapman HD, Jeffers TK, Williams RB (2010) Forty years of monensin for the control of coccidiosis in poultry. Poult Sci 89(9):1788–801. Epub 2010/08/17. doi: 10.3382/ps.2010-00931 20709963 10.3382/ps.2010-00931

[28] Lee HJ, Cho SS, Simkhada JR, Yoo JC (2009) Monoclonal antibody production and immunochemical detection of polyether antibiotics. Arch Pharm Res 32(3):437–41. Epub 2009/04/24. doi: 10.1007/s12272-009-1318-7 19387589 10.1007/s12272-009-1318-7

